# Supplementary material for: Complexome profiling of the Chlamydomonas psb28 mutant reveals TEF5 as an early PSII assembly factor
Source: Plant Cell. 2025 Mar 17;37(6):koaf055. doi: 10.1093/plcell/koaf055 (PMC12164590; doi:10.1093/plcell/koaf055)
Supplement: koaf055_Supplementary_Data [file koaf055_supplementary_data.zip › psb28_interactive.zip!/plots/intensity/qualityPlots/peptides/nad2_Cre-2.g801491.t1.p1.html]

### Cre-2.g801491.t1.p1

Trivial name: nad2  
SeqCoverage [median]: 2.90  
Mass: 42.38 kDa  
  
 

| Sequence | MissedCleavage | Mass [Da] | wtIntensity | muIntensity | Score |
| --- | --- | --- | --- | --- | --- |
| SLLLYLSTAPK | 0 | 1204.71 | 3885.54 | 2196.01 | 132.01 |
|  |  |  | 3885.5 | 2196.0 |  |
